# Optimizing renal replacement therapy for patients who need extracorporeal membrane oxygenation: cross-talk between two organ support machines

**DOI:** 10.1186/s12882-019-1602-9

**Published:** 2019-11-13

**Authors:** Kianoush Kashani, Marlies Ostermann

**Affiliations:** 1 Department of Medicine, Division of Nephrology and Hypertension, Mayo Clinic, Rochester, MN USA; 2 Department of Medicine, Division of Pulmonary and Critical Care Medicine, Mayo Clinic, 200 First Street SW, Rochester, MN 55905 USA; 3 King’s College London, Guy’s & St Thomas’ Hospital, London, UK

**Keywords:** Extracorporeal membrane oxygenation (ECMO), Acute kidney injury, Dialysis, Continuous renal replacement therapy

## Abstract

Following a substantial increase in the utilization of extracorporeal membrane oxygenation (ECMO) during the last decade, its associated benefits and complications, including acute kidney injury have become more apparent. Acute kidney injury requiring dialysis during the ECMO treatment is very common and is associated with adverse outcomes. Cross talk between ECMO and dialysis equipment has been debated in the literature in order to enhance the quality of dialysis and avoid its potential adverse events.

Na et al. recently published the results of a prospective experiment by using three different methods for integration of the continuous renal replacement therapy device into the ECMO circuit. In this experiment, the investigators showed that by using three different connection strategies between continuous renal replacement therapy device and ECMO and the utilization of three separate structures of pressure control lines, the dialyzer lifespan could be optimized.

In this commentary, following a brief review of the ECMO and dialysis devices history and cross talk, we discuss the findings by Na et al. and provide additional insights for future investigations.

## Background

Extracorporeal membrane oxygenation (ECMO) was first tried in patients with respiratory failure in the early 1970s by Donald Hill, a surgeon in the San Francisco area [1]. Following initial successful implementation and reasonable results based on case series, a randomized clinical trial funded by the National Institute of Health resulted in a negative conclusion regarding its performance [2]. Subsequently, the use of ECMO for adult patients with cardiorespiratory failure became limited to a very small number of programs. This changed after the CESAR trial in 2009, which showed that patients with severe potentially reversible respiratory failure who were transferred to ECMO centers had a better survival without severe disability compared to patients who were treated in non-ECMO centers [3]. This study, combined with important advances in technology, led to a significant increase in the utilization of ECMO for patients with cardiac and/or respiratory failure [4, 5].

### Main text

Acute kidney injury (AKI) is a common complication in patients receiving ECMO due to multiple injurious mechanisms, including underlying inflammation, hypoperfusion, and exposure to nephrotoxins [5, 6]. The incidence of AKI among ECMO patients has been reported between 30 and 70% [7, 8]. Renal replacement therapy (RRT) is frequently required (9% among patients with respiratory failure and 12% among individuals who needed ECMO for cardiac failure) [5]. As the majority of patients are hemodynamically unstable, continuous renal replacement therapy (CRRT) is the modality of choice. Although each type of extracorporeal support can be delivered independently using separate venous access, many institutions integrate the CRRT device into the ECMO circuit [6]. Finding an efficient and safe method for connecting a CRRT device into an ECMO circuit is the focus of the current investigation.

The pressure within the ECMO circuit is variable depending on the location before or after the centrifugal pump. The pump generates a negative pressure in the inlet arm (− 20 to -100 mmHg) in order to pull a predefined blood volume from the patient (usually 3–6 l per minute). After the pump, however, the intra-circuit pressure is positive to ensure adequate blood flow into the oxygenator and eventually into the patient. In comparison, CRRT devices are set up to be connected to venous pressure 0 to + 20 mmHg [9] (equivalent to central venous pressure) and have built-in pressure alarms. The ECMO pump is able to generate pressures as high as 600 mmHg, which is higher than the safety limit of the ECMO circuit (around 300 mmHg) [10] and significantly higher than that of CRRT devices.

When integrating the CRRT circuit into the ECMO circuit, an appreciation of the intra-circuit pressures and the potential risks (including air entrapment, flow disturbance, hemolysis) is essential to avoid potentially life-threatening complications. Connecting the CRRT device to the ECMO circuit may result in high- or low-pressure alarms depending on where the CRRT connections are made in relationship with the ECMO pump. To avoid high access pressures in the CRRT inlet line, the options are to connect the access line to the ECMO circuit pre-pump or to decrease the CRRT blood flow rates [9]. Similarly, to avoid high pressures in the return line (CRRT outlet line), connecting the access line to the ECMO circuit pre-pump could be considered. Other options are to apply adjustable clamps to the RRT circuit [9]. Using clamps and pressure control lines could potentially reduce the number of alarms and improve the health of the dialyzer, and, therefore, its lifespan, but results in potential changes in the blood flow and an increased risk of turbulences and hemolysis.

Na et al. [11] analyzed a prospectively collected dataset of ECMO patients who received CRRT using three different methods for integrating the connecting the CRRT device into the ECMO circuit. In all patients, the CRRT inlet (access) line was connected to the ECMO post-pump line (i.e., positive-pressure), and the CRRT outlet (return) line was attached to the ECMO pre-pump line (i.e., negative pressure). The authors differentiated between three cohorts based on whether pressure control lines were used and explored the impact on the CRRT filter lifespan. In the first group, the CRRT device was connected to the ECMO circuit using Luer Lock connections alone without pressure control devices (*N* = 16). Among patients in the other two groups, the CRRT device was integrated into the ECMO circuit using Luer Lock connections with an additional pressure control line on the inlet (*N* = 36) or both the inlet and outlet lines (*N* = 118), respectively. While the investigators did not find any difference in platelet count, lactate dehydrogenase, and hemoglobin values among the 3 groups, they reported significantly higher CRRT circuit lifespan when a pressure control line was applied to both, the inlet and outlet of the CRRT circuit (avoiding high positive or negative pressures within the CRRT circuit) in comparison with the other two groups. In addition, they observed significantly lower mortality among patients with double pressure control lines compared to the other two groups.

This study provides substantial knowledge regarding the practical aspects of ECMO-CRRT cross-talk and shows the impact of simple interventions on the cost of treatment and filter lifespan, and potentially their effects on the clinical outcomes. Importantly, the authors observed that patients who required RRT following initiation of ECMO started RRT shortly after initiation of ECMO, i.e., median of 1 (0–2) day. Naturally, patients who require ECMO have significant organ failure (at least respiratory or cardiovascular) and potentially a high incidence of volume overload. In this patient cohort, the gap between demand and endogenous capacity of kidney function widens fast, which may warrant the initiation of RRT very early in the course.

Na et al. showed that simple interventions to reduce the pressures within the dialysis circuit were associated with an increased lifespan of the dialyzer, and potentially lower costs. However, it is not clear how to explain the reduction in mortality among patients who had pressure control devices on both sides of the CRRT machine. In addition, a higher incidence of metabolic acidosis at the initiation of RRT was noted in the first group compared to the other two groups, which suggests that overall clinical practice may have changed during the course of the study.

## Conclusions

While the study by Na et al. provides excellent insights regarding the impact of ECMO-CRRT cross-talk on the quality of care and potential patient outcomes, it does not offer a generalizable approach for the integration of ECMO and CRRT devices as there is significant variability between the programs regarding the type of devices, patient populations, indications of extracorporeal life support and CRRT, and finally the prescriptions used for each of the device. However, the analysis offers a path to a better understanding of this cross-talk and provides options on how to overcome challenges related to intra-circuit pressures in order to optimize treatment.

## Data Availability

Not applicable.

## Abbreviations

AKI: Acute Kidney Injury
CESAR trial: Conventional ventilatory support versus extracorporeal membrane oxygenation for severe adult respiratory failure trial
CRRT: Continuous Renal Replacement Therapy
ECMO: Extracorporeal Membrane Oxygenation
RRT: Renal replacement therapy
